# Pandemic Influenza and Hospital Resources

**DOI:** 10.3201/eid1311.070103

**Published:** 2007-11

**Authors:** Raoul E. Nap, Maarten P.H.M. Andriessen, Nico E.L. Meessen, Tjip S. van der Werf

**Affiliations:** * University Medical Center Groningen, Groningen, the Netherlands

## Abstract

Using estimates from the Centers for Disease Control and Prevention, the World Health Organization, and published models of the expected evolution of pandemic influenza, we modeled the surge capacity of healthcare facility and intensive care unit (ICU) requirements over time in northern Netherlands (≈1.7 million population). We compared the demands of various scenarios with estimates of maximum ICU capacity, factoring in healthcare worker absenteeism as well as reported and realistic estimates derived from semistructured telephone interviews with key management in ICUs in the study area. We show that even during the peak of the pandemic, most patients requiring ICU admission may be served, even those who have non–influenza-related conditions, provided that strong indications and decision-making rules are maintained for admission as well as for continuation (or discontinuation) of life support. Such a model should be integral to a preparedness plan for a pandemic with a new human-transmissible agent.

## Abstract 2

Even during the peak of a pandemic, all patients requiring intensive care can be served.

## Abstract 3

Keywords: influenza pandemic, health care facilities, surge capacity, research

The threat of an avian influenza A (e.g., subtypes H5N1, H7N7) pandemic has forced healthcare authorities and health services to draft and discuss preparedness plans (*1*–*5*). The responsibility for management of the national and regional risks due to pandemic influenza was underscored by the outbreak of avian influenza (H7N7) in 2003 in the Netherlands, which led to culling one third of domestic poultry (including 30 million chickens), with 1 human casualty, a veterinary surgeon who died from acute lung injury after infection with the virus (*6*,*7*). The increasing pandemic threat of influenza A (H5N1) is reflected by 291 cases of human disease reported to the World Health Organization (WHO) as of April 11, 2007, with 172 human deaths (*8*). Because the question is not whether a pandemic will occur but, rather, when (*9*), policymakers have been urged to take action in preparedness planning.

Preparing for an influenza pandemic is difficult for healthcare systems because of many uncertainties. Strikingly little knowledge has been obtained from the scattered cases of avian influenza in humans (*10*).

In influenza patients admitted to an intensive care unit (ICU), severe disease may develop with a sepsis-like pattern with a proinflammatory cytokine storm (*11*), but it is unknown what percentage of patients fall ill after acquiring the virus (attack rate) and what percentage require hospital admission and, subsequently, ICU admission. Attack rate, hospital and ICU length of stay, and death rate can only accurately be factored in after a new virus has emerged (*3*). Therefore, almost all assumptions in the models published to date have drawn on the knowledge obtained from the large 20th-century pandemics (*12*–*14*). In summary, a model for preparedness of the healthcare system should be highly adaptable and flexible to factor in new information emerging in the early stages of the pandemic.

The University Medical Center Groningen (UMCG) is a large tertiary care university hospital covering ≈12% of the total Dutch population and ≈30% of the total surface area of the Netherlands. Under Dutch law, UMCG has an important role in the event of an avian influenza pandemic, not only for the patient population that it serves but also as a regional coordinating center (*15*). Training courses that emphasized the need to enhance collaboration and communication for pandemic influenza were held with regional and municipal health authorities, general practitioners, and representatives of all hospitals in the northern region. We present a model, similar to models by Anderson et al. (*16*) for Australia and New Zealand and Menon et al. for England (*14*). We show that increased hospitalization in combination with healthcare worker (HCW) absenteeism will have a substantial, but in our model manageable, effect on hospital and ICU bed occupancy. Furthermore, we discuss the choices to be made for ongoing, non–influenza-related emergencies during an influenza pandemic and the effect of enhancing the contingency plans already in place. Although surge capacity of hospital resources is typically limited (*1*), we explored whether, under specified assumptions and appropriate planning and training, a pandemic is manageable.

## Methods

We used FluSurge 2.0 (*17*) and a computer model in an Excel file developed by one of the authors to calculate the impact of an influenza pandemic in the Netherlands on hospital admission and occupancy rate of all ICU beds (i.e., those with facilities for mechanical ventilation). Data on population (≈1.7 million) and age distribution (Table 1) were obtained from publicly available sources. The age distribution in the Dutch population data were provided in 5-year groupings, and we therefore converted these data to an even distribution to allow for calculations with the FluSurge program (*14*). Data on total hospital beds, ICU beds, and number of nurses and their full-time equivalents were obtained from publicly available sources (*18*). ICU capacity was also obtained from reports from hospital administrators during training sessions for pandemic influenza in May 2006, organized by the public health authorities in the region. These data on reported ICU capacity were discussed during a semistructured telephone interview with ICU medical staff in August 2006. Using these data, we estimated the regular bed capacity and maximal surge capacity. Data on the impact of a pandemic influenza on healthcare services were adopted from the National Institute for Public Health and the Environment (RIVM) (*19*,*20*). RIVM presented tables for 25% and 50% disease attack rates, representing best and worst case scenarios. From these tables we calculated the 30% attack rate (percentage of the population that becomes ill) by linear transformation. A 30% attack rate is the most likely scenario, according to the Centers for Disease Control and Prevention, and is defined as the most likely scenario by RIVM.

**Table 1:** Age distribution of inhabitants of 3 northern provinces in the study, the Netherlands

Province	Age range, y					Total, all ages
	0–15	16–24	25–44	45–64	>65	
Groningen	99,065	72,714	164,371	151,590	86,818	574,558
Friesland	125,174	70,397	174,768	172,600	99,665	642,604
Drenthe	92,241	45,885	127,674	136,915	81,212	483,927
Total	316,480	188,996	466,813	461,105	267,695	1,701,089

We also calculated, within the model, the total number of patients admitted to the hospitals at each point in time during the pandemic. We defined the first day (day 0) as the moment that WHO declares human-to-human transmission (phase IV or V in the current WHO phase of pandemic alert). We took into account the time each patient occupies a hospital or ICU bed (range 8–15 days), on the basis of experience with patients admitted to ICU with a diagnosis of pneumonia or sepsis. Finally, we incorporated estimated risk of death per patient, reducing the number of admitted patients at any one time. Because the data of the RIVM are in week blocks, we evenly distributed the number of hospital admissions and the proportion of deaths across the week days.

In our calculations, we also factored the effect of treatment (within 48 hours of infection) with antiviral medication on the spread and the impact of the pandemic, although the exact effect size is still uncertain (*14*,*21*). Antiviral medication is assumed to reduce the total number of hospital admissions by 50% and death rate by ≈30%.

In addition, we incorporated in the model the probable absenteeism of HCWs either due to illness or to care duties at home or in individual social environments. We assumed that HCWs will become ill at a rate similar to that of the general population. We extrapolated national population data of illness and deaths to the total number of HCWs in our HCW database.

Finally, we incorporated the effect of strict treatment decisions at the patient level on the peak occupancy rate of ICU beds. We applied a 48-hour restriction of treatment time at the ICU for patients occupying an ICU bed. We focused our preparedness plan on adults, assuming an outbreak pattern similar to that of Spanish flu (*22*) and severe acute respiratory syndrome (SARS), in which adolescents and adults accounted for most cases.

## Results

**Figure 1:**
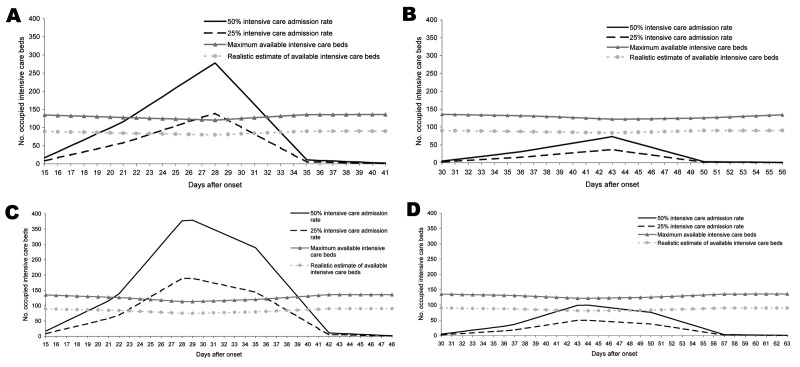
A) 30% attack rate and mean length of stay of 8 days without antiviral medication, pandemic period 9 weeks; B) 30% attack rate and mean length of stay of 8 days with antiviral medication, pandemic period 14 weeks; C) 30% attack rate and mean length of stay of 15 days without antiviral medication, pandemic period 9 weeks; D) 30% attack rate and mean length of stay of 15 days with antiviral medication, pandemic period 14 weeks.

We present the impact of a pandemic with new human-transmissible influenza on hospital resources in the northern part of the Netherlands. Using the figures of the RIVM, and assuming a 30% cumulative disease attack rate, we estimated that ≈12% of the population will consult a general practitioner (Table 2). The percentage of persons triaged for hospital admission is 0.3%. We assumed excess deaths among these selected patients, some 50% of whom may require mechanical ventilation (Figure 1). In the northern part of the Netherlands 5,629 regular hospital beds are available. The hospitals in this region have a total of 30% (non–influenza-related) acute care, which would leave 3,940 regular hospital beds that could be made available for influenza-related hospital admissions. If the attack rate reaches a maximum of 50% with a mean length of stay of 15 hospital days per patient, without any intervention, this would lead to a peak of 1,227 occupied regular hospital beds, which would suffice for influenza-related acute care. Therefore, we centered our calculations around the peak occupancy of intensive care beds. We calculated the number of hospital admissions per week, spread evenly across 7 days in the respective week, and we subtracted the number of deaths, also evenly spread across the week. We assumed that 25%–50% of total hospital admission patients would require some form of mechanical ventilator support, and we provide calculations for the extremes of our estimates. On the basis of results from a semistructured telephone interview with ICU medical staff of the hospitals in the 3 northern provinces, a maximum of 136 (of a total of 200) ICU beds could be dedicated to influenza-related acute-care patients. We estimate that 90 ICU beds will be made available in a short period. In the scenario of no additional intervention, if the full capacity of all 136 ICU beds is used, with an attack rate of 30%, 25% ICU admissions, and a mean length of stay of 8 days, we would have a shortage of 3 ICU beds at day 28 after onset, when we expect the pandemic to peak. This shortage in ICU capacity is exacerbated with any increase in hospital length of stay or ICU length of stay.

**Table 2:** Avian influenza impact for 3 northern provinces in the Netherlands*

**Week**	**Days**	**No. patients**	**General practitioner consultations**	**Hospital admissions**	**Deaths**
0	1–7	0	0	0	0
1	8–14	105	11	0	0
2	15–21	4,694	515	11	0
3	22–28	145,898	16,559	315	84
4	29–35	347,288	44,699	977	420
5	36–42	25,935	3,696	95	74
6	43–49	578	84	0	0
7	50–56	11	0	0	0
8	57–63	0	0	0	0
9	64–70	0	0	0	0
Total		524,507	65,562	1,397	578

*30% attack rate, pandemic period 9 weeks.

**Figure 2:**
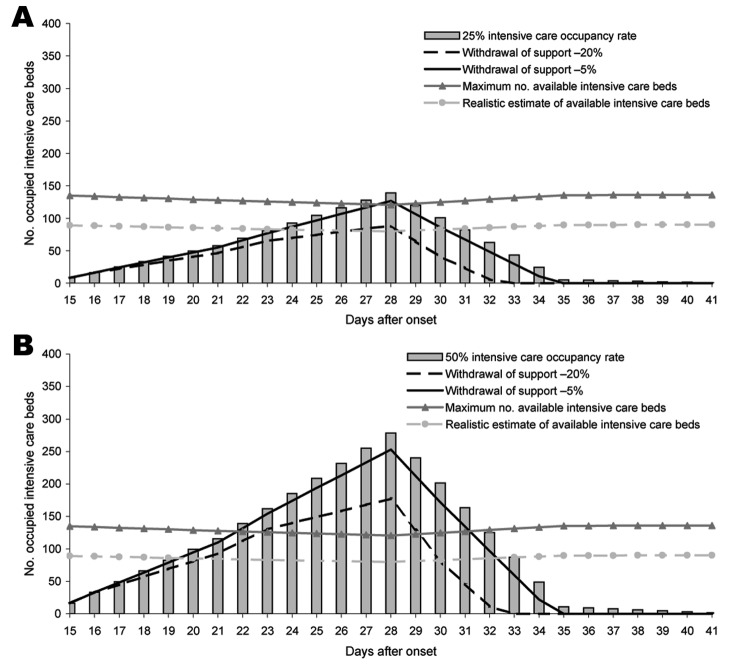
A) Effect of intensified treatment decision (25% intensive care unit [ICU] admission rate, mean length of stay of 8 days) without antiviral medication, pandemic period 9 weeks; B) effect of intensified treatment decision (50% ICU admission rate, mean length of stay of 8 days) without antiviral medication, pandemic period 9 weeks.

HCWs would become ill in the pandemic in proportion to the attack rate in the general population, and we illustrated the impact of HCW absenteeism on loss of ICU bed capacity for all presented scenarios (Figures 1, 2). Furthermore, we visualized the effect of intensified treatment decisions on the occupancy of ICU beds (Figure 2). For this situation, we used the representative case scenario estimate data, i.e., 30% attack rate and a mean length of stay of 8 days, and show the effect of intensified treatment decision resulting in reduction of ICU occupancy by 5% and 20%. Intensified treatment decision was defined as discontinuation of mechanical ventilation after 48 hours, based on ample consultations within ICU teams and with partners and next of kin of patients that the patients are deemed to have no realistic hope for recovery. Finally, we made sensitivity analyses, with changing assumptions within the model; this additional material is presented in an online Technical Appendix (available from www.cdc.gov/EID/content/13/11/zzz-Techapp.pdf).

## Discussion

We provide calculations for hospital bed and ICU capacity for an influenza pandemic made for 1 region in the Netherlands showing that even during the peak of the pandemic, hospital facilities can continue to provide adequate healthcare service to the public. As a novel element we include calculations for HCW absenteeism. We have not considered potential erosion of professionalism with increased absenteeism due to fear and panic among staff or due to staff members’ caring for sick family members. Although morale was high during the SARS outbreak in Singapore and Toronto (*23*), some examples of strained professional behavior have been reported (*24*). We believe that erosion of professionalism and morale may be partly preventable by implementing effective protection for HCWs (*25*,*26*), with appropriate training to comply with protocols for personal protection. For a new pandemic, the important issues to factor in are magnitude and duration, calculation of staff shortages, and the limited capacity to call in external resources.

We show that an influenza pandemic can be managed, even allowing emergency care for non–influenza-related acute cases, especially when firm decision-making rules are followed and antiviral therapy is used. Without withdrawing or withholding life support to those deemed to have no realistic chance of survival, the system is bound to collapse (Figure 2). With appropriate patient management, however, adequate healthcare can be provided even during the peak of the pandemic. We recognize the ethical impact this has on the clinicians and nurses who have to make these decisions. Many clinicians now realize that end-of-life decisions are an integral part of healthcare (*27*) and can be considered independent of any specific religious background or culture (*28*). ICU staff in the Netherlands have been trained to take charge of decision processes about foregoing life support in the ICU (*27*). They are aware of potential difficulties in communicating with members of the ICU team, including medical, nursing, and technical staff in decisions at the end of life. The challenge during an outbreak of pandemic influenza will be in orchestrating and implementing these decisions under extreme time pressure. Relatives of patients as well as team members may need more time than available to accept that some patients on life support who are not responding to treatment will not recover. Some may insist on continuation of support, although it would be unwise and possibly disrespectful to these patients to continue futile treatment and unfair to others who might have been saved if those resources had been available. A generous and time-consuming approach may not apply under the anticipated extreme conditions of pandemic influenza (*27*).

Decision-making rules have to be adapted to real-time information updates obtained during the course of the pandemic, and briefings and exchange of information throughout the pandemic crisis are pivotal. Existing guidelines and protocols such as the Pneumonia Severity Index or its modification recommended by the American Thoracic Society or the British CURB-65, propagated by the British Thoracic Society, may not apply fully but can be used initially to guide management of patient treatment (*29*). Our overall assessment that an influenza pandemic with assumptions described here can be managed at the level of healthcare institutions clearly contrasts with the sobering and daunting analysis presented for ICU capacity in the United Kingdom or Australasia (*14*,*16*).

There are limitations to our analysis. We based our model on incomplete and sometimes conflicting or inconsistent information on the impact of an influenza pandemic. We assume that more reliable data will only become available when the pandemic is in progress. The effect of antiviral medications, vaccination campaigns, and, for instance, closure of schools and airports may alter the key characteristics of the pandemic, all having the effect that onset is delayed and that the course is more protracted, with a much lower peak (*12*). Even a less-than-perfect vaccine might have a tremendous impact on the course of the pandemic. Stockpiling of influenza A (H5N1) virus is now being considered in order to produce vast quantities of vaccine despite the limited protection capacity against the new virus.

The need for surge capacity of hospital resources is more dependent on the combination of excess hospital admissions and length of stay than on the mere number of hospital admissions. In the Netherlands, stockpiling of oseltamivir has been implemented, both for the public at large and for healthcare facilities and HCWs working on the frontlines during the influenza pandemic. Stockpiling of antimicrobial agents to combat secondary bacterial pneumonia is yet another important logistic challenge (*30*).The small percentage of patients admitted to hospital in our model (based on past experiences) implies that relatively small increases in admittance rate will have a huge impact on hospital resources requirement.

Extensive exposure may lead to seroconversion to avian influenza viruses, as has been shown for influenza A (H11N9) virus among waterfowl hunters and wildlife professionals (*31*). The policy in the Netherlands since this was discovered has been that all persons involved in culling should wear respiratory masks, gowns, gloves, and eye protection. Although the effectiveness of these precautions has not been prospectively tested, they might protect persons from contracting respiratory viral disease. In our hospital protocol for management of patients of new pandemic influenza and of other high-risk respiratory pathogens, we have included extensive measures to separate these patients from other patients and focus on the protection of staff (*1*). Adherence to similar protocols has been shown to protect HCWs caring for patients with SARS (*26*). In summary, we recommend using and updating the model presented here, or similar models, as an integral part of a preparedness plan and as a management tool for contingency of pandemic influenza.
